# Changes in Sperm Morphology, Morphometry, and Motility from the Epididymis to the Vas Deferens in Rheas (*Rhea americana*, Linnaeus, 1758)

**DOI:** 10.3390/ani13091483

**Published:** 2023-04-27

**Authors:** Luana G. P. Bezerra, Andréia M. Silva, Artur P. Jurema, Maiko R. T. Dantas, Ana G. Pereira, Moacir F. Oliveira, Pierre Comizzoli, Alexandre R. Silva

**Affiliations:** 1 Laboratory of Animal Germplasm Conservation, Department of Animal Sciences, Universidade Federal Rural do Semi-Árido (UFERSA), Mossoró 59625-900, Brazil; luana_grasielly@yahoo.com.br (L.G.P.B.); andreia.m.silva@hotmail.com (A.M.S.);; 2 Smithsonian National Zoo and Conservation Biology Institute, Washington, DC 20008, USA

**Keywords:** bird reproduction, ratites, sperm morphology, epididymis, vas deferens

## Abstract

**Simple Summary:**

The rhea (*Rhea americana*) is nearly threatened in nature, while playing important ecological and economic roles. Therefore, the study of its reproductive biology is necessary to provide information that will serve as a basis for its conservation through natural breeding and reproductive biotechnologies. We characterized rhea spermatozoa and analyzed changes that occur in the cells during the transit between the epididymis to the vas deferens (based on sperm motility, morphology, morphometry, and ultrastructure). We reveal, for the first time, that rhea spermatozoa undergo an increase in size and motility throughout their transit in the spermatic pathways.

**Abstract:**

The objective was to characterize morphological, morphometric, and ultrastructural changes in rhea spermatozoa between the epididymis and the vas deferens. Sperm samples were collected from the reproductive tracts of seven adult individuals and evaluated for sperm characteristics using brightfield microscopy as well as ultrastructural features using scanning electron microscopy (SM). Mean sperm count tended to increase in the vas deferens (378.0 ± 135.0 × 10^6^) compared to the epididymis (201.0 ± 77.4 × 10^6^). Percentages of motile sperm grew from 37.0 ± 4.9% in the epididymis to 58.5 ± 7.7% in the vas deferens. The proportion of normal spermatozoa was 75.6 ± 1.8% and most common defects were bent tails (9.7 ± 0.9%). However, these proportions were not different between epididymis and vas deferens. SM analysis revealed further features of rhea spermatozoa. Normal rhea spermatozoa were threadlike with an acrosome (0.95 ± 0.0 µm), head (7.53 ± 0.01 µm), midpiece (2.08 ± 0.01 µm), and tail (30.7 ± 0.06 µm). Lengths of sperm acrosome, head, midpiece, and tail were longer in the vas deferens compared to the epididymis. Our findings suggest that rhea spermatozoa undergo a maturation process during the passage from the epididymis to the vas deferens.

## 1. Introduction

Rheas (*Rhea americana*) are birds of substantial size endemic to biomes in South America. These birds play a vital role as seed dispersers and serve as prey for large predators within their native ecosystems [1]. Regrettably, rheas are classified as near threatened globally by the International Union for the Conservation of Nature [2]. The destruction of their eggs by agricultural machinery or burning in their natural habitat is one of the leading causes of population decline [2]. Additionally, rheas are frequently hunted for their highly valued meat in the gastronomic market [3], feathers and eggs utilized in decorative item manufacturing, and fat with noted therapeutic properties that include nutritional, analgesic, healing, and cosmetic effects [4].

Given the aforementioned, the need to develop programs for in situ and ex situ conservation of rheas is pressing. To achieve this, a comprehensive understanding of the species’ biological parameters is crucial, with particular emphasis on reproductive aspects. This knowledge is essential for both captive animal propagation and the development of assisted reproductive technologies [5]. Nonetheless, research on rhea reproduction remains scarce [6,7]. While the development of spermatozoa in rheas’ testes has been previously documented [8], basic parameters concerning changes in sperm morphology along the spermatic pathways, such as the epididymis and vas deferens, are yet to be explored. This understanding is of particular importance as spermatozoa in birds are produced in the testes and then matured and stored in the epididymis and vas deferens [9].

The analysis of sperm morphology has been acknowledged as a crucial approach in the field of andrology due to the potential use of sperm cell dimensions as biological indicators of environmental effects. Moreover, it provides valuable insights into the processes of spermatogenesis and the reproductive and evolutionary strategies of sperm species [10]. Additionally, the assessment of sperm morphology not only serves as an indicator of gamete quality, but also enables the evaluation of the male reproductive system, since semen quality is indicative of the testes, seminiferous, and epididymal tubule health [11]. High frequencies of abnormal spermatozoa or a high incidence of a single defect can lead to reduced fertility [12]. Moreover, differences in the sizes and shapes of cells or their nuclei in ejaculates may be associated with various subpopulations of mature spermatozoa that have a direct impact on fertilization capacity, as reported in mammals [13].

Sperm morphology analysis can be conducted through various methods, including the use of smears stained with different dyes and observed under brightfield microscopy, which offers the advantage of being a low-cost and rapid technique [14]. This method has enabled the examination of sperm morphology in different avian species, such as turkey (*Meleagris gallopavo*) [15], gander (*Anser anser*) [16], rooster (*Gallus gallus domesticus*) [17], ostrich (*Struthio camelus*) [18], emu (*Dromaius novaehollandiae*) [14], golden eagle (*Aquila chrysaetus*) [19], peregrine falcon (*Falco peregrinus peregrinus/brookei*), and gyrfalcon (*Falco rusticolus*) [20]. Nevertheless, this technique has limitations in the scope of information it provides. Therefore, the use of electron microscopy offers higher precision and detailed visualization of biological structures at a nanometric scale, providing a more comprehensive, accurate, and reliable description [21].

Given the insufficient information regarding the reproductive aspects of rheas, this study aimed to elucidate the alterations in the morphology, morphometry, and ultrastructure of sperm as they transit from the epididymis to the vas deferens, thereby shedding light on the storage mechanisms in this species of ratite bird.

## 2. Materials and Methods

The ethics committee of the Universidade Federal Rural do Semi-Arido (UFERSA) approved the experimental protocols as well as the animal care procedures adopted (Process no. 23091.001423/2020-8). In addition, experiments were approved by the Brazilian Ministry of the Environment (SISBIO No. 73638-1). The reagents used in this study were obtained from Sigma-Aldrich (St. Louis, MO, USA).

### 2.1. Animal

The animals used were obtained from the Center for the Multiplication of Wild Animals (CEMAS/UFERSA; IBAMA register No. 12.492-0004), located at 5°11′ S and 37°20′ W, at an average altitude of 16 m in a region characterized by a semi-arid tropical climate, presenting Köppen climatic classification as dry and very hot, with an average annual temperature of 27.4 °C. To conduct the experiment, we used the reproductive tracts of animals that were part of the CEMAS population surplus, which, as a scientific breeding facility, is authorized to slaughter them with the sole purpose of carrying out different experiments that will contribute to the acquisition of important information about the species. Seven adult rheas, aging 2.9 ± 0.6 years old and with an average weight of 24.2 ± 1.5 kg, were selected. Sperm collections took place during the breeding seasons, corresponding to the 3 months following the end of the rainy season in the region. The paddocks had a dimension of 20.00 × 10.00 m, where 2 males were placed for 6 females. The rheas were fed with concentrated feed based on corn, wheat bran, and soybean, with seasonal fruits being occasionally provided. Water was provided ad libitum.

### 2.2. Recovery of Spermatozoa

The animals were captured manually and euthanized based on the protocol used by Pollock et al. [22]. In summary, after mechanical restraint, the animals were weighed and submitted to the anesthetic procedure, with an association of intramuscular pre-anesthetic medication of xylazine hydrochloride at 1 mg/kg (Xilazin^®^ 2%, Syntec, Pharmaceutical Technology Applied to Veterinary Medicine, São Paulo, Brazil) and ketamine hydrochloride 15 mg/kg (Quetamina^®^, Vetnil, Sao Paulo, Brazil). After reaching the sedation stage, the animals were anesthetized with 150 mg/kg thiopental (Thiopentax; Cristalia, São Paulo, Brazil). When the anesthetic plane was reached, they were euthanized with intracardiac potassium chloride at 2.56 mEq/kg (Potassium Chloride^®^ 19.1%, Equiplex, Goiânia, GO, Brazil). The animal’s death was confirmed after identifying the cardiopulmonary arrest.

After euthanasia, the complex consisting of the testicles, epididymis, and vas deferens was collected, wrapped in gauze moistened with 0.9% saline at 38 °C, placed in Becker, and transported in a thermal box to the laboratory for processing. Subsequently, they were dissected, washed externally with physiological solution at 38 °C, weighed, and measured. To obtain spermatozoa, the flotation technique was used. Briefly, the whole epididymis and vas deferens were separated and sliced with a scalpel in a Petri dish containing saline at 38 °C. After 5 min in a static position, the tissues were removed, and the suspension was evaluated [23].

### 2.3. Initial Evaluation of Recovered Sperm Sample

The recovered volume was measured using pipettes and graduated tubes. An aliquot of the sample containing spermatozoa (5 μL) was diluted in 10% buffered formalin (995 μL) and the sperm concentration was estimated using a Neubauer counting chamber [24]. The number of recovered spermatozoa was measured, multiplying the sperm volume and concentration. Subsequently, subjective sperm motility (0–100%) and vigor (0–5) were assessed on a semen aliquot deposited on a glass slide previously heated to 37 °C under a brightfield microscope (100x, 400x; Nikon Eclipse E200, Nikon Instrument, Tokyo, Japan) by two different observers. Subjective motility was graded from 0 (absence of movement) to 100% motile spermatozoa, while vigor was determined on a scale considering 0 = no movement, 1 = tail movements but no sperm progression, 2 = only circular sperm movements, 3 = a large percentage of sperm moving but not in a rectilinear fashion, 4 = a large percentage of sperm showing straight, but not vigorous movement, and 5 = a large percentage of sperm showing vigorous, straight, progressive movement [20].

### 2.4. Sperm Morphological Analysis

To evaluate the morphological characteristics of the spermatozoa, slides were prepared as follows: a 5 µL aliquot of the recovered sample from the epididymis and vas deferens was separately incubated with 45 µL of the Bengal Rose dye (Dinâmica^®^ Química Contemporânea LTDA, Indaiatuba, Brazil) (0.58 g sodium citrate + 0.8 mL formaldehyde + 0.3 g rose bengal + 20 mL distilled water). Subsequently, 10 µL of the incubated sample was removed and deposited on a slide that was covered with a coverslip and sealed. A hundred sperm cells were analyzed under brightfield microscopy (100x) (Nikon Eclipse E200, Nikon Instrument, Tokyo, Japan), being classified as morphologically normal or with alterations. The description of the morphological defects found in rhea spermatozoa was conducted in accordance with what was previously reported for domestic fowl [17,25].

### 2.5. Sperm Morphometric Analysis

The Bengal Rose-stained smears were also used for the morphometric evaluations. For measurements, random fields of the slide were photographed using brightfield microscopy (Leica ICC50 HD, Wetzlar, Germany) connected to image software Leica Las EZ software version 2.0.0. One hundred cells were analyzed using photomicrographs, measuring spermatozoa structures such as head, midpiece, and tail separately using the ImageJ^®^ software (ImageJ Software, Wayne Rasband, National Institute of Health, Bethesda, MD, USA) [26].

### 2.6. Scanning Electron Microscopy—SM

For ultrastructural analysis, the methodology of Bezerra et al. [27] was used with adaptations; rhea sperm from the epididymis and vas deferens were separately fixed in Karnovsky’s reagent (4% paraformaldehyde and 2.5% glutaraldehyde in 0.2 M phosphate buffer, pH 7.2) at 27 °C. Samples were centrifuged at 800× *g* for 10 min to form a pellet, then three washes of 5 min each with 0.2 M phosphate buffer were performed. Subsequently, the samples were post-fixed in 1% osmium tetroxide diluted in water, distilled, and dehydrated in alcohol baths at increasing concentrations (50, 70, 90, and 100%) for 10 min each. The pellets were broken up and a drop of the suspension was placed on a glass coverslip and air-dried. Coverslips were mounted on stubs using carbon tape. For metallization, the stubs were placed in a metallizer and metallized with a 20 nm gold layer for later observation with a scanning electron microscope (Tescan Vega3; Tescan Analytics, Fuveau, France). A total of 60 cells from each organ was observed.

### 2.7. Statistical Analysis

Data were expressed as mean and standard error. Analyses of homoscedasticity and normality were performed using the Shapiro–Wilk and Levene tests. The Kruskal Wallis test was used and comparisons between epididymis and vas deferens samples were performed using the Dwass–Steel–Cristcholow–Fligner (DSCF) test. Furthermore, differences between different individuals were also checked using the DSCF test for multiple comparisons. Results were considered significant when *p* < 0.05. The analyses were performed using the Jamovi Software version 2.3.19 (Sydney, Australia).

## 3. Results

### 3.1. Characteristics of the Rhea Reproductive Organs

**Figure 1:**
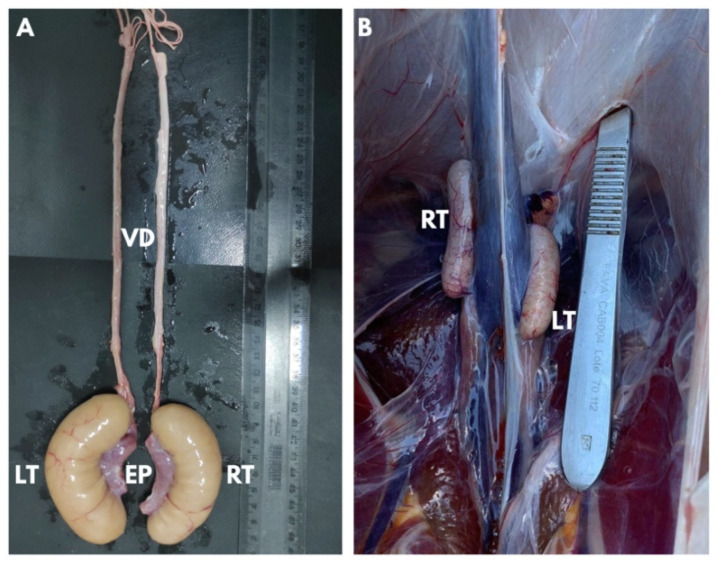
Testicles of rheas (*Rhea americana*) reared in semi-arid region, Brazil. (**A**) Normal rhea testicles. (**B**) Atrophied testes in the coelomic cavity. VD—vas deferens, EP—epididymis, RT—right testicle, and LT—left testicle.

Rhea testes were located in the intra-abdominal region (coelomic cavity) dorsal to the gastrointestinal tract and ventral to the kidneys. Of the seven rheas collected, viable sperm samples were obtained from only five animals. The normal testicles observed in those five individuals were cream-colored and cylindrical in shape, with rounded, bean-shaped ends (Figure 1A). The epididymis was red in color, adjacent to the testicles (Figure 1A). The vas deferens were cream-colored with a sinuous and then straight shape in the final portion (close to the cloaca). The average weight of the viable right testicles was 27.4 ± 5.6 g, and 30.6 ± 8.9 g for the viable left testicles. The average length was 63.6 ± 10.5 mm and 69.7 ± 10.7 mm for the right and left testicles, respectively. The mean diameter of the right testicle was 19.5 ± 1.0 mm, and 22.3 ± 2.6 mm for the left one. No difference was observed between the weight and the size of the right and left testes (*p* > 0.05).

Two rheas presented small testicles (Figure 1B) weighing an average of only 3.83 ± 1.5 g. From these atrophied testes, it was not possible to obtain samples with sufficient sperm concentrations to conduct evaluations. Data from these two individuals were excluded from all assessments, including testicular measurements.

### 3.2. General Characteristics of the Recovered Sperm Samples

There were no differences between left and right organs regarding the volume, sperm concentration, sperm motility, or vigor (Table 1). However, combined sperm motility (left and right organs) increased (*p* < 0.05) in the vas deferens compared to the epididymis.

The group of male rheas that were studied formed a relatively homogeneous population, with no differences being identified between individuals for any of the parameters evaluated in this study, except for sperm morphometry, which differed between individuals (*p* < 0.05).

### 3.3. Overall Sperm Morphology and Morphometry

**Figure 2:**
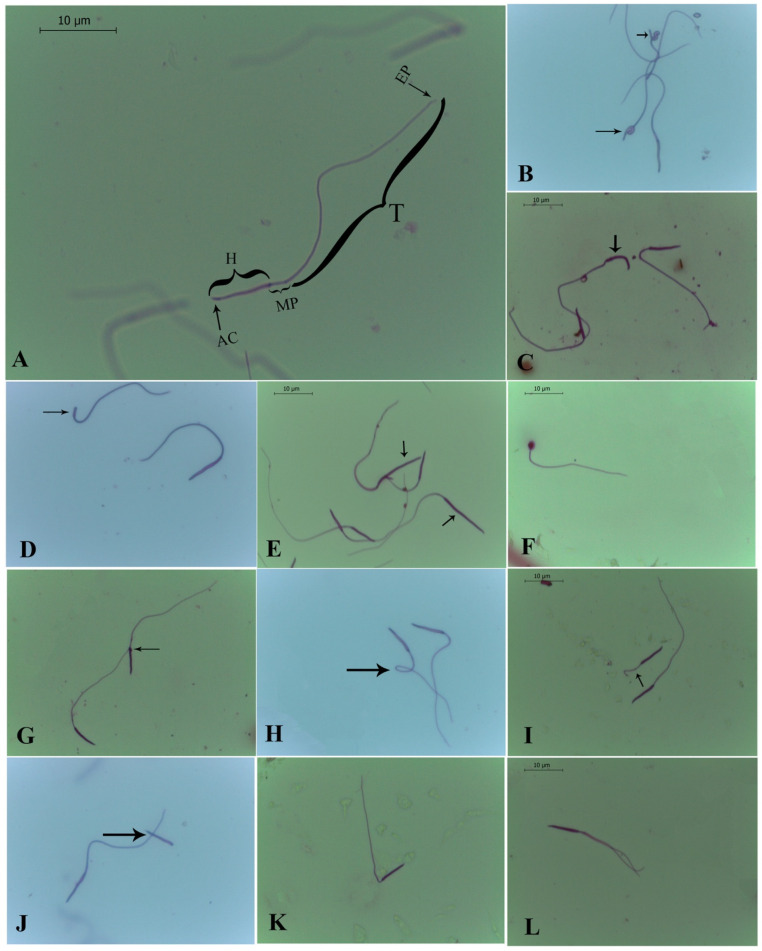
Morphology of rhea (*Rhea americana*) spermatozoa from the epididymis and vas deferens stained with Bengal Rose. (**A**) Normal sperm with intact acrosome. (**B**) Bent head (black arrows). (**C**) Hook-shaped head. (**D**) Blunt head (black arrow). (**E**) Macrocephaly (black arrows). (**F**) Spermatid-like sperm. (**G**) Cytoplasmic droplet (black arrow). (**H**) Bent tail (black arrow). (**I**) Broken tail (black arrow). (**J**) No tail (black arrow). (**K**) Bent midpiece. (**L**) Two tails. AC—acrosome, H—head, MP—midpiece, T—tail, EP—endpiece.

Normal rhea sperm cells had a cylindrical shape with a well-defined head, an acrosome in the anterior portion of the head, a middle piece, and a tail (Figure 2A). The tail was divided into the main portion and the final portion (Figure 2A). 

Morphological analysis of the rhea sperm is reported in Table 2. Overall, there were no differences in morphological features between right and left spermatic pathways). From the epididymis to the vas deferens, an average of 75.6 ± 1.8% normal spermatozoa was observed (Figure 2A; Table 3). The most common abnormality was the bent tail (Figure 2H) followed by the macrocephaly (Figure 2E) (Table 3). Heads of these macrocephalic spermatozoa were around 11.8 ± 0.15 µm in length, which corresponds to about 4.3 µm longer than spermatozoa with normal morphology.

Regarding sperm morphometry (Table 3), there was an increase (*p* < 0.05) in the lengths of the acrosome, midpiece, tail, total sperm, and head width between sperm retrieved from the epididymis and sperm cells retrieved from the vas deferens.

### 3.4. Sperm Ultrastructure

**Figure 3:**
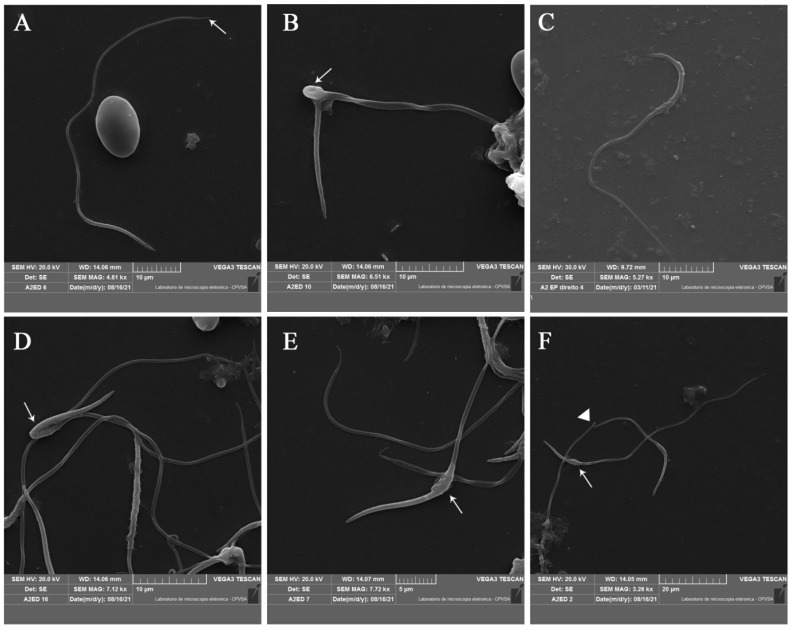
Scanning electron microscopy of rhea (*Rhea americana*) spermatozoa from the epididymis. (**A**) Normal sperm with endpiece (white arrow). (**B**) Macrocephalic sperm, with two tails and bent midpiece (white arrow). (**C**) Sickle-shaped head. (**D**) Bent head. (**E**,**F**) Swelling at the base of the head (white arrows). (**F**) Broken tail (white triangle).

Normal sperm cells were long and narrow (Figure 3A). The various segments (acrosome, nucleus, midpiece, and tail) could be easily distinguished, although the transition in some cases between regions was not observed. The acrosome formed the anterior tip of the sperm head, having the base clearly demarcated from the nucleus, which constituted the longest segment of the head. The fact that the midpiece had a rougher surface helped to distinguish between the head and this structure, presumably due to the presence of the mitochondrial sheath in this region. The midpiece gradually tapered towards the visibly thinner tail. The tail was in fact the longest segment of the sperm and ended abruptly in a final thin and short piece that was more clearly seen through SM, as it had been poorly visualized in brightfield microscopy due to its small thickness (Figure 3A). We observed macrocephalic and two-tailed spermatozoa (Figure 3B), and it was found that in fact, two-tailed spermatozoa had only one intermediate piece, with two tails that intertwined (Figure 3B). Spermatozoa with a sickle-shaped head (Figure 3C), bent heads (Figure 3D), swelling at the base of the head (Figure 3E,F), and broken tails (Figure 3F) were also observed

## 4. Discussion

This study is the first to report on the morphological characteristics of rhea sperm along the spermatic pathways. A clear sequence of alterations in sperm morphology and morphometry from the epididymis to the vas deferens was observed, corresponding to events associated with sperm maturation and storage. The evaluated parameters show great repeatability among individuals, which did not present significant differences among themselves, except for sperm morphometry.

The testis size, length, and diameter of normal rheas observed in this study were comparable to those reported in a previous study conducted in the same biome [7]. Out of the seven animals utilized, two were unable to produce sperm samples due to atrophied testes without any sperm production despite being in their reproductive season. Due to the social relationship observed in rheas [28], we suspect that there is a decrease in testosterone production in dominated males. Thus, sexual characteristics such as increased testicular volumes would be suppressed as observed in spotted antbirds (*Hylophylax n. naevioides*) [29] and canaries (*Serinus canaria*) [30].

The number of spermatozoa recovered from the vas deferens was lower than that obtained by Góes et al. [6] following cloacal massage (2.24 × 10^9^ sperm). It is evident that the collection method can impact the success of sperm retrieval, but other factors like weather and diet can also influence sperm production [31].

The motility of sperm collected from the vas deferens was comparable to that observed in rhea ejaculates, as reported by Góes et al. [6], with a mean percentage of 61.11 ± 11.54%. Additionally, the motility of sperm obtained from the vas deferens was significantly higher than that of sperm collected from the epididymis. Avian sperm progressively acquire the abilities required for fertilization during their passage through the male genital tract before ejaculation [32]. Tingari [33] proposed that the vas deferens in domestic birds was equivalent to the mammalian epididymal body and tail, which suggests that the vas deferens may play a role in the maturation and acquisition of sperm motility and fertilizing ability. Although the underlying mechanisms of these functional changes remain unknown, previous studies have indicated that proteins secreted in the epididymis and vas deferens bind to the sperm surface, protecting spermatogenic cells against oxidative damage and contributing to the sperm functions required for fertilization [9].

Despite the good staining, visualization of the distal portion of the sperm tail was challenging due to its small diameter, and the tail was better observed using SM. This technique allowed not only a confirmation of the findings obtained by brightfield microscopy, but also an appreciation of the richness of details observed in the different regions that compose the sperm cell. 

Regarding the morphological analysis of spermatozoa, it is worth mentioning that our results were within the normal range. Specifically, the minimum percentage of morphologically normal sperm in an ejaculate should be in the range of 70–80% to achieve optimal fertility, as suggested by Kuster et al. [34]. Among the sperm defects observed in rheas, the tail region was the most commonly affected, followed by the head and, finally, the middle piece. Regarding sperm head defects, various forms of bent head were observed, ranging from soft to acute folds. These defects were also observed in SM in the present work as well as in emus [35]. This defect may be associated with sample processing, but reports suggest that it may originate from spermiogenesis due to defective, incomplete, or absent chromatin condensation, especially near the base of the head, making this area more susceptible [35]. Some rhea spermatozoa had a curved hooked head, while others had blunt heads or small cylindrical heads. However, these defects require further investigation to better understand their origin and effect on the fertility of the species. Round-headed spermatozoa were also observed in rheas, which some authors have classified as spermatids [17]. These appear to be the result of defective spermiogenesis, specifically involving failure of nuclear elongation, possibly due to the defective formation of the circular mantle responsible for initiating and maintaining nuclear elongation in several bird species [35].

The prevalence of macrocephalic sperm in the rhea was high. The occurrence of large-headed sperm has also been documented in great bustards [36], ostriches [14], and emus [35]. It has been suggested that the enlarged heads of avian sperm contain more than haploid genetic material [36].

The cytoplasmic droplets in rhea spermatozoa exhibited a bulbous shape, situated between the base of the head and the beginning of the midpiece. The presence of cytoplasmic droplets in sperm may indicate a deficiency in sperm maturation [35]; however, this defect was very uncommon in rheas.

Tail defects were the most abundant in rhea spermatozoa, and they have significant implications for sperm motility [11]. However, this may have been influenced by sample processing. It is known that after mating, sperm are deposited in the vagina and undergo an intense selection process, resulting in only a limited number of sperm entering the sperm storage tubules (SSTs). The mechanism of sperm selection in this process is still poorly understood; however, it is believed that sperm motility may play an important role in sperm uptake in SSTs [37].

Proportions of spermatozoa with broken tails increased in the vas deferens compared to the epididymis in rheas. Tailless sperm were evidenced only by the presence of the head, which may have resulted from processing or disruption of the implantation fossa. The prevalence of two-tailed spermatozoa in rheas was consistent with previous findings in emus [38]. This defect was also observed through SM in macrocephalic sperm. In rheas, spermatozoa possessed a single intermediate piece, which was wider than usual, potentially indicating an increased number of centrioles. Multiflagellate spermatozoa seem to originate from two sources, namely incomplete cytokinesis and abnormal centriolar duplication [38]. The low incidence of this defect in mammals suggests a minor effect on fertility [39]. The prevalence of two-tailed spermatozoa in rheas was only approximately 1%, indicating a minor impact on semen quality and fertility.

As observed through morphometric analysis, rhea spermatozoa display similar dimensions to those of other ratite birds such as the ostrich (69.59 ± 0.31 µm) [40] and emu (67.64 ± 3.13 µm) [14], despite appearing smaller. From the measurements, it was observed that the average size of the acrosome in the spermatozoa retrieved from the vas deferens of rheas was larger compared to those in the epididymis. This phenomenon has been linked to sperm maturation in mammals, where spermatozoa stored in the epididymal cauda exhibit more developed acrosomes than those from the caput of the epididymis [41]. It is noteworthy that although avian spermatozoa do not undergo capacitation in the female reproductive tract, they still undergo acrosomal reaction [42], making it necessary for the acrosome to mature during development.

Additionally, there was an increase in the midpiece length of the spermatozoa retrieved from the vas deferens compared to those from the epididymis of rheas. This increase is possibly due to the development of the mitochondrial sheath during sperm maturation, which provides potential energy required for motility [43]. Moreover, flagellum length also increased, as it is positively correlated with midpiece length in both birds and mammals [44].

Because rheas are a near-threatened species, we were ethically obligated to minimize the number of animals used in the present studies. We emphasize, however, that the data from the evaluation of the five animals are still highly relevant, given the scarcity of information in this species. Indeed, it is worth noting that similar sample sizes were used in previous studies to describe sperm parameters of other wild species, from critically endangered Southern muriqui (*Brachyteles arachnoides*) [45] and Bornean orangutans (*Pongo pygmaeus*) [46] to species of least concern, such as six-banded armadillos (*Euphractus sexcinctus*) [47] and crab-eating foxes (*Cerdocyon thous*) [48]. Therefore, numerous directions for future studies will come from these initial results as the description of functional aspects of the rhea sperm, such as the kinetic parameters determined by computer analysis, mitochondrial activity, and chromatin status. Furthermore, it is known that birds are internally fertilized, and sperm are stored in the female for an extended period [37]. It would also be interesting to study sperm parameters from samples collected directly from the sperm reservoir in females.

## 5. Conclusions

In summary, it can be deduced that rhea spermatozoa exhibit morphological resemblance to gametes of other ratite birds, despite their smaller size and thread-like shape. Furthermore, there was a substantial increase in the dimensions of these spermatozoa during their transport from the epididymis to the vas deferens, owing to their maturation process. Our findings also showed that there was an increase in sperm motility along the transit from the epididymis to the vas deferens. These observations carry significant implications for advancing our comprehension of reproductive mechanisms in rheas and can facilitate the design of conservation strategies.

## Figures and Tables

**Table 1:** Characteristics of sperm samples recovered from the epididymis and vas deferens of adult rheas (*Rhea americana*). Values are expressed as mean ± SEM from 5 individuals.

	Right Epididymis	Left Epididymis	Right Vas Deferens	Left Vas Deferens	Left + Right	
					Epididymis	Vas Deferens
Recovered volume (mL)	1.4 ± 0.2	1.1 ± 0.1	1.0 ± 0.1	1.1 ± 0.1	2.5 ± 0.3	2.0 ± 0.3
Concentration (×10^6^ sperm/mL)	96.0 ± 31.9	68.0 ± 27.5	170.0 ± 39.4	222.0 ± 88.2	82.0 ± 28.7	196.0 ± 60.4
Number of sperm recovered (×10^6^)	126.0 ± 48.1	75.5 ± 30.3	152.0 ± 39.6	233.0 ± 106.0	201.0 ± 77.4	378.0 ± 135.0
Sperm motility (0–100%)	36.0 ± 5.1	38.0 ± 4.9	58.0 ± 7.18	59.0 ± 9.0	37.0 ± 4.9 ^a^	58.5 ± 7.7 ^b^
Vigor (0–5)	2.4 ± 0.4	2.6 ± 0.5	3.2 ± 0.2	3.4 ± 0.2	2.5 ± 0.5	3.3 ± 0.2

^a, b^ Different letters mean significant difference between the two columns (*p* < 0.05).

**Table 2:** Percentage of normal sperm and proportion of different morphological abnormalities in rhea (*Rhea americana*) epididymis and vas deferens. Values are expressed as mean ± SEM from 5 adult individuals.

	Epididymis (Left + Right)	Vas deferens (Left + Right)
Normal sperm (%)	75.7 ± 2.0	75.4 ± 3.3
Head defects (%)		
Bent head	2.4 ± 0.5	5.1 ± 1.8
Hook-shaped head	1.2 ± 0.3	0.7 ± 0.2
Blunt Head	0	4.0 ± 0
Macrocephalic	2.2 ± 1.4	9.5 ± 9.0
Spermatid-like	2.0 ± 1.5	0.5 ± 0
Midpiece defects (%)		
Bent midpiece	1.4 ± 0.2	2.9 ± 1.5
Cytoplasmic droplets	2.2 ± 0.8	0.5 ± 0
Tail defects (%)		
Bent tail	11.2 ± 1.2	8.3 ± 1.3
Broken tail	5.2 ± 0.9 ^a^	2.7 ± 0.8 ^b^
No tail	2.6 ± 0.6	5.6 ± 1.3
Two tails	1.3 ± 0.3	1.1 ± 0.3

^a, b^ Different letters mean significant difference between the two columns (*p* < 0.05).

**Table 3:** Rhea (*Rhea americana*) sperm morphometry in samples recovered from the epididymis and vas deferens. Values are expressed as mean ± SEM from 5 individuals.

	Epididymis (Left + Right)	Vas Deferens (Left + Right)
Acrosome length (µm)	0.93 ± 0.01 ^a^	0.97 ± 0.01 ^b^
Head length (µm)	7.52 ± 0.02 ^a^	7.54 ± 0.02 ^a^
Head width (µm)	0.57 ± 0 ^a^	0.59 ± 0 ^b^
Midpiece length (µm)	2.05 ± 0.01 ^a^	2.12 ± 0.04 ^b^
Tail length (µm)	30.3 ± 0.10 ^a^	31.0 ± 0.09 ^b^
Total length (µm)	40.0 ± 0.11 ^a^	40.8 ± 0.11 ^b^

^a, b^ Different letters mean significant difference between the two columns (*p* < 0.05).

## Data Availability

The data presented in this study are available on request from the corresponding author.
